# MicroRNAs targeted mTOR as therapeutic agents to improve radiotherapy outcome

**DOI:** 10.1186/s12935-024-03420-3

**Published:** 2024-07-04

**Authors:** Shahram Taeb, Davoud Rostamzadeh, Seyed Mohammad Amini, Mohammad Rahmati, Mohammad Eftekhari, Arash Safari, Masoud Najafi

**Affiliations:** 1 Department of Radiology, School of Paramedical Sciences, Guilan University of Medical Sciences, Rasht, Iran; 2 Department of Immunology, University of Connecticut Health Center, Farmington, CT USA; 3 Radiation Biology Research Center, Iran University of Medical Sciences, Tehran, Iran; 4 Department of Medical Biotechnology, Faculty of Paramedicine, Guilan University of Medical Sciences, Rasht, Iran; 5 Department of Radiology, Ionizing and Non-Ionizing Radiation Protection Research Center (INIRPRC), School of Paramedical Sciences, Shiraz University of Medical Sciences, Shiraz, 71439-14693 Iran; 6 Radiology and Nuclear Medicine Department, School of Paramedical Sciences, Kermanshah University of Medical Sciences, Kermanshah, Iran; 7 Medical Biology Research Center, Institute of Health Technology, Kermanshah University of Medical Sciences, Kermanshah, Iran; 8 Medical Technology Research Center, Institute of Health Technology, Kermanshah University of Medical Sciences, Kermanshah, Iran

**Keywords:** MicroRNAs, mTOR, Therapeutic agents, Radiotherapy

## Abstract

MicroRNAs (miRNAs) are small RNA molecules that regulate genes and are involved in various biological processes, including cancer development. Researchers have been exploring the potential of miRNAs as therapeutic agents in cancer treatment. Specifically, targeting the mammalian target of the rapamycin (mTOR) pathway with miRNAs has shown promise in improving the effectiveness of radiotherapy (RT), a common cancer treatment. This review provides an overview of the current understanding of miRNAs targeting mTOR as therapeutic agents to enhance RT outcomes in cancer patients. It emphasizes the importance of understanding the specific miRNAs that target mTOR and their impact on radiosensitivity for personalized cancer treatment approaches. The review also discusses the role of mTOR in cell homeostasis, cell proliferation, and immune response, as well as its association with oncogenesis. It highlights the different ways in which miRNAs can potentially affect the mTOR pathway and their implications in immune-related diseases. Preclinical findings suggest that combining mTOR modulators with RT can inhibit tumor growth through anti-angiogenic and anti-vascular effects, but further research and clinical trials are needed to validate the efficacy and safety of using miRNAs targeting mTOR as therapeutic agents in combination with RT. Overall, this review provides a comprehensive understanding of the potential of miRNAs targeting mTOR to enhance RT efficacy in cancer treatment and emphasizes the need for further research to translate these findings into improved clinical outcomes.

## Introduction

MicroRNAs (miRNAs) are small non-protein-coding RNA molecules made up of nearly 18 nucleotides [1]. MiRNAs are estimated to contribute about 1–5% of the human genome and generate more than 30% of protein-coding genes [2]. The first miRNA was identified in 1993, and then a great deal of findings has shown that they may operate as tumor inhibitors or cause tumorigenesis [3–5]. Moreover, miRNAs have been found to have a pivotal effect on gene regulation, particularly when they are attached to control the numerous cell and metabolic processes, as well as being a major member of the knockdown mechanism in most eukaryotic [6]. Some findings have shown that miRNAs have a critical role in the biological activities of different cancers [7, 8]. A connection between miRNAs and cancer cells have been established, with miRNAs being identified as a possible strategy that might enhance cancer therapy techniques by restoring or suppressing miRNA activity [9].

The mammalian target of rapamycin (mTOR) and the signaling networks are essential for preserving cell homeostasis by regulating a variety of biological activities such as cell proliferation and immune response. The definition of the mTOR kinase substrate characterization relates to which associate of the protein it binds with. mTORC1 is formed of mTOR and four specific proteins called raptor, mLST8, PRAS40, and DEPTOR. mTORC1 regulates cap-dependent translation initiation, which is required to produce several oncogenic proteins like cyclin D1, c Myc, Mcl-1, and Snail. mTORC2 includes mTOR, Rictor, mLST8, DEPTOR, mSin1, and protor and phosphorylates Akt, serum and glucocorticoid-inducible kinase (SGK), and protein kinase C (PKC) [10]. In contrast to mTORC1, the biological roles of mTORC2, especially ones associated with oncogenesis control other than cytoskeleton and cell survival, have not been thoroughly characterized, even though mTORC2 is implicated in the positive modulation of cancer progression [11, 12]. Currently, only a few details are revealed about the upstream controls of the mTORC2 center; also, mTORC1 acts as a conjunction site for the phosphoinositide 3-kinase (PI3K)/Akt and mitogen-activated protein kinase (MAPK)/MEK/ERK mechanism, which is usually excited in malignancies [13].

MiRNAs can potentially impact the mTOR mechanism in different methods, including interacting with mTOR, affecting the members of mTOR complexes, and affecting either the negative or positive essential upstream modulator of mTOR, which consequently impacts the efficacy of mTOR activation [14, 15]. Given the importance of the mTOR pathway in immune response regulation, miRNA-mediated mTOR pathway modulation can alter the efficacy of immunological responses and a broad range of immune-related diseases [16, 17]. Based on the preclinical findings, the blending of mTOR modulators with radiotherapy (RT) can decrease the development of solid tumors through simultaneous anti-angiogenic and anti-vascular impacts [18, 19]. In previous studies, we have examined the role of RT doses alone on cancer and stem cells in the tumor microenvironment [20–23]. In this study, we summarized the last findings of the miRNAs targeted mTOR, which is used as a therapeutic agent to improve RT outcomes.

## The mTOR signaling network

In a typical environment, mTOR is an essential modulator of cell proliferation and division, which is known. Nevertheless, mTOR which is improperly stimulated in tumor cells sends out signals that trigger tumor cells to proliferate, spread, and infiltrate healthy tissues located nearby or far from it [24]. The PI3K/phosphate and fungal homology mutation in chromosome 10 (PTEN)/AKT/ TSC axis is the most important regulator of mTORC1, and gene mutations in this system might cause cancer [25]. Furthermore, in most malignant tumors, PTEN activity is frequently suppressed by epigenetic, genetic, and post-transcriptional changes to activate the PI3K/Akt/mTOR mechanism [26, 27]. mTORC1 is comprised of multiple proteins including, mTOR, Raptor (regulator-associated protein of mTOR), PRAS40 (proline-rich Akt substrate, 40 kDa), Deptor, mLst8 (mammalian lethal with Sect. 13 protein 8), Tti1, and Tel2. mTORC2 is composed of mTOR, Rictor, mSin1 (mammalian stress-activated protein kinase-interacting protein 1), Protor1/2, Deptor, mLst8, Tti1, and Tel2 [28, 29].

It has been shown that mutation in the PTEN gene results in the deregulation of the PI3K/PTEN signaling pathway in hepatic cell carcinoma [30]. Additionally, mutation of the PTEN gene causes the production of an immunosuppressive molecule, programmed cell death protein (PD-1/CD279) 1, which triggers immunosuppression and accelerates tumor development, progression, and metastasis [31]. Hyperactivation of PI3K/PTEN/Akt/mTOR axis is implicated in liver cancer cell proliferation and migration by activating matrix metallopeptidase 9 (MMP9) [32]. Likewise, the PI3K/Akt/mTOR activation has been revealed to regulate cancer cell growth and survival in different human cancers [33]. It has been shown that a mutation in the liver kinase B1 (LKB1) gene or an external growth signal might stimulate mTORC1 [34]. mTORC1 suppresses the function of the ring finger protein 168 (RNF168) protein and increases its decomposition through phosphorylating the 60th serine which results in diminished ubiquitination of histone H2A and H2A histone family member X (H2AX) following DNA damage, inhibiting the response to DNA damage and decreasing genome stability, promoting malignant cell transformation and cancer development [35]. Furthermore, Rheb is also a GTPase that connects and stimulates mTORC1 when GTP is supplied. Ubiquitination of Rheb was controlled with growth factor indications. Ubiquitination of Rheb inhibits its kinase activity which stimulates Rheb attachment to TSC2, resulting in the suppression of mTORC1 kinase activity [36].

**Fig. 1:**
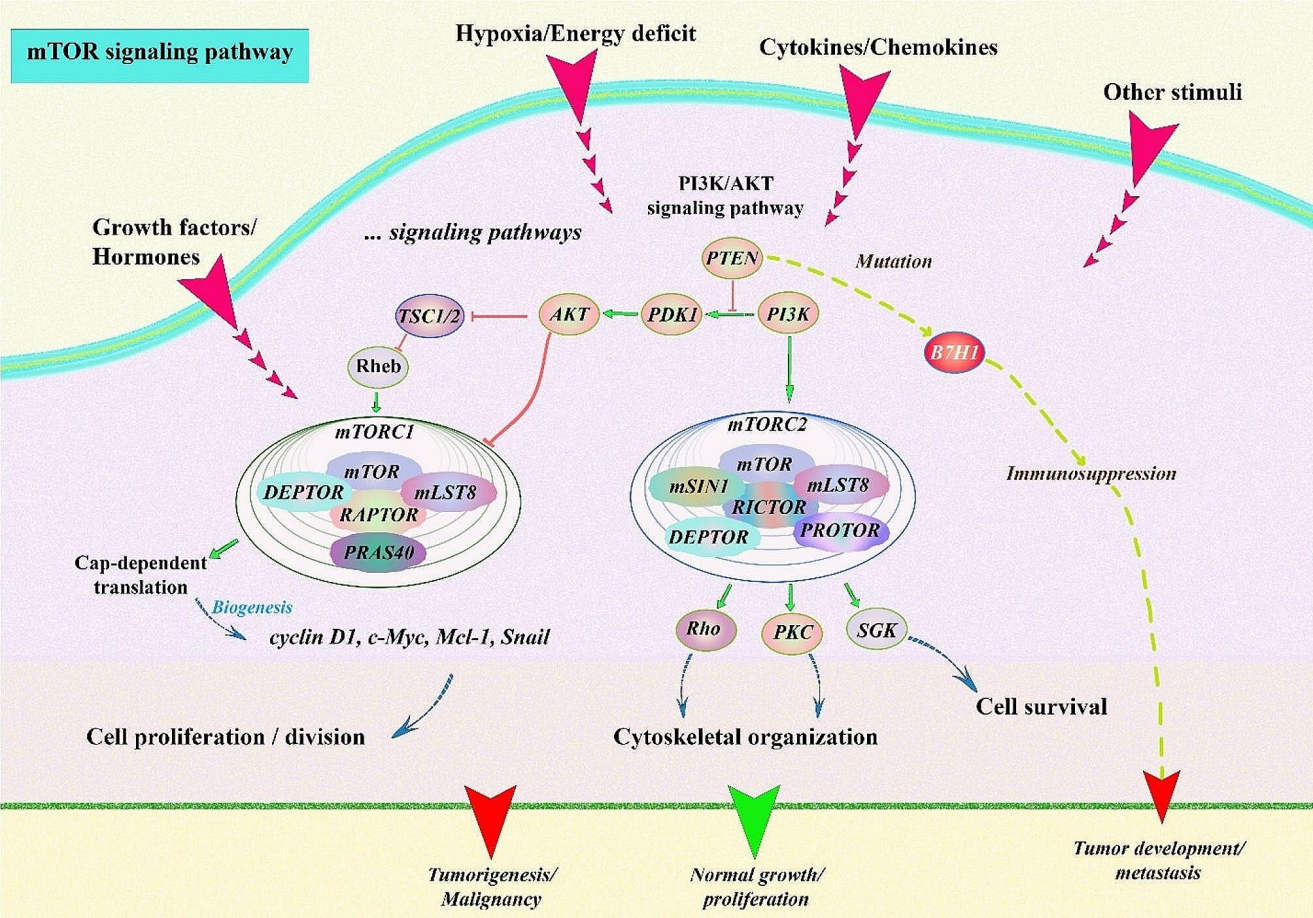
A comprehensive map of the mTOR signaling network

Similar to mTORC1, mTORC2 activity is associated with tumor cell development and progression [37]. OTU deubiquitinase 7B (OTUD7B) diminishes the ubiquitination amount of G protein β-like (GβL), prevents GβL binding to SIN1, resulting in mTORC2/AKT signaling upregulation and conversely the downregulation of mTORC1 activity. This increases carcinogenesis by partly activating AKT oncogenic activity [38, 39]. On the other hand, the ubiquitin ligase tumor necrosis factor (TNF) Receptor Associated Factor 2 (TRAF2) increases the amount of GβL ubiquitination. Additionally, Ras mutations increase the activities of mTORC2 kinase by attaching to mTOR of mTORC2 and mitogen-activated protein kinase-associated protein 1 (MAPKAP1), therefore affecting cell cycle programs that promote proliferation [40, 41]. Hence, mTOR is constantly activated in tumors to keep tumor cells proliferating and surviving and it serves a crucial function in tumor cell biology (Fig. 1).

## MiRNAs

miRNAs are small non-coding RNA molecules, typically 21–23 nucleotides in length, that play crucial roles in post-transcriptional gene regulation. They are involved in various biological processes, including development, cell proliferation, differentiation, and apoptosis. By binding to the 3’ untranslated region (UTR) of target messenger RNAs (mRNAs), miRNAs can inhibit translation or induce mRNA degradation, thereby controlling gene expression [42].

### miRNAs biogenesis

Synthesis of miRNA is regulated by RNA polymerase II which produces a hairpin precursor defined as a primary miRNA, which is subsequently processed by endoribonucleases Dicer (in the cytoplasm) and Drosha (in the nucleus). Most miRNAs are produced by intergenic non-coding sequences; although they may be encoded in either a sense or antisense path in exonic or intronic domains, and hence their promoters could synchronize their transcription independently [43, 44]. miRNAs are classified according to their sequence matching and activity, and they might be found in the genome as single parts or in groups. A single miRNA is expected to adversely modulate many unique target mRNAs [45]. Meanwhile, it should be emphasized that the cellular targets and function of the majority of miRNAs have not been figured out.

Canonical miRNA production starts with the generation of an extended primary miRNA via RNA pol II. Clustered miRNAs could be encoded using a single polycistronic primary miRNA as a transcription unit. Due to having 7-methyl diguanosine triphosphate (7 M-GpppG) as a 5′-cap in the formation of primary miRNA and canonical miRNAs, they have a similar structure [46, 47]. So, when the primary miRNA has been transported to the cytoplasm, the loop is degraded by the dicing function of the RNAse III enzyme in combination with TRBP2 (dsRNA-binding protein). Regarding, primary miRNA is transported to the RNA-induced silencing complex and handled by the Argonaute protein. Following the break of the passenger strand via Argonaute protein, developed miRNA could lead the miRNA-induced silencing complex to reach complementary mRNA strands [48]. This developed miRNA could suppress gene expression through base matching of its target mRNA and subsequently control the occurrence of target mRNAs as well as the regulatory method [49]. Most of the time, translational suppression of transcripts is caused by precise matching between the miRNA and the targeted mRNA, which results in mRNA degradation facilitated by the RNA-induced silencing complex. Furthermore, the probability of mistakes in miRNA-mRNA pairing implies that a single miRNA can attach to multiple mRNAs [47, 50]. Gene expression regulated by miRNAs begins in cytoplasmic granules including ribonucleoproteins (RNPs), known as mRNA processing bodies (P-bodies), which are made up of mRNA decay-related components and miRNAs [51, 52]. As a result, P-bodies serve as sites for performing of the cytoplasmic mRNA in a post-transcriptional direction. P-bodies are involved in the destruction, preservation, and monitoring of mRNAs as well as downregulation processes based on RNA observed in many cell lines [53, 54].

### miRNA functions

**Fig. 2:**
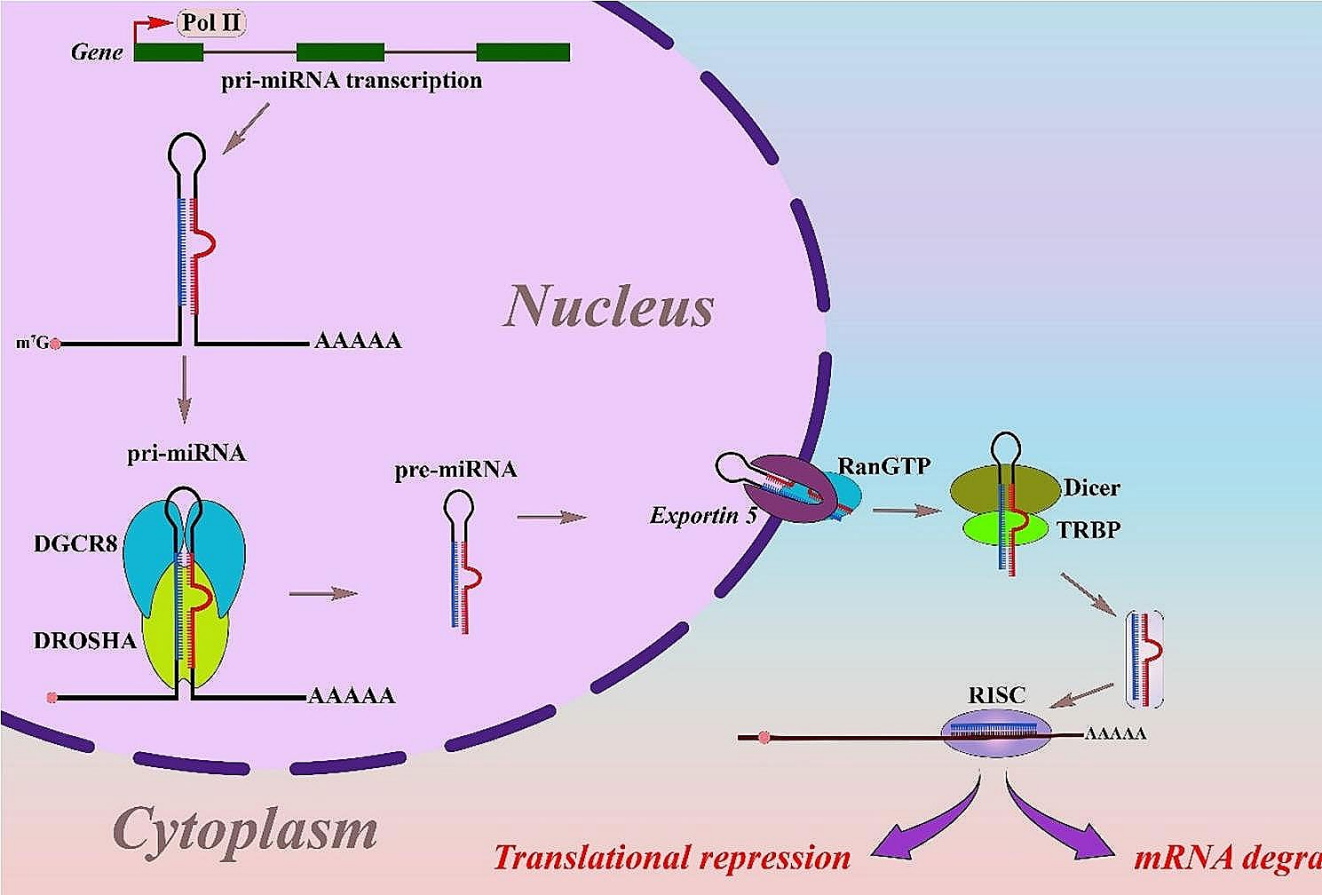
Overview of the microRNAs Biogenesis and Functions

In the genome, miRNA coding genes are found in both intergenic and intron regions, and they are organized into groups where a single main transcript creates many miRNAs [55, 56]. Two stages are required to produce human miRNAs, which are made of small RNA duplexes derived from long endogenous transcripts. Additionally, Drosha and Dicer are ribonuclease III enzymes involved in cleavage [57]. Pre-miRNA is produced by the Drosha, whereas miRNA duplexes are produced by the Dicer [58]. One strand of such a duplex forms the RNA-induced silencing complex, which is defined as the miRNA guide strand. As RNA-induced silencing complexes are generated, the miRNA guide strand attaches to mRNA 3` UTRs based on a base-matching pattern [57]. Gene expression is controlled by miRNAs by connecting to mRNA targets and causing decomposition or suppression of translation, depending on the complementarity between the miRNA and mRNA 30 UTR. Moreover, complete complementarity leads to mRNA breakdown, whereas incomplete complementarity prevents mRNA translation. Several sites in the human genome are compatible with binding areas for miRNAs, including gene coding sequences, gene promoters, and the 5-UTR [59]. (57). In extracellular contexts such as serum, plasma, blood, urine, and saliva, miRNAs can be detected in small quantities, but the majority are found inside cells [60]. Furthermore, miRNAs are being investigated as indicators for pathophysiological disorders and as targets for novel targeted therapies [61]. Several miRNAs have the potential to influence osteogenic differentiation in both favorable and unfavorable ways [62]. Therefore, a specific miRNA could be used as a negative or positive modulator gene and transcription factor (TF) in such a situation [63] (Fig. 2).

## Crosstalk between mTOR signaling and miRNAs in cancer

The crosstalk between mTOR signaling and miRNAs in cancers has emerged as a significant area of research. The mTOR pathway is a central regulator of cell growth and metabolism, while miRNAs play a crucial role in post-transcriptional gene regulation. Understanding the interplay between these two regulatory mechanisms can provide valuable insights into the molecular mechanisms underlying cancer development and progression.

### miRNAs regulate mTORC1 signaling pathway

miRNA activity interacts with upstream and downstream components of mTOR signaling pathways as well as with mTOR itself, which affects the fundamental physiological processes, such as cell growth, migration, and apoptosis [64, 65]. It has been shown that miRNAs-199a inhibits the growth of liver cancer, glioma, and endometrial cancer by suppressing mTOR expression. Conversely, miRNAs − 205 promotes non-small cell lung cancer growth by downregulating PTEN expression, which inhibits TOR signaling pathways through the PI3K-Akt pathway, and TSC1/TSC2 [66]. The miRNAs − 218 promotes apoptosis in oral squamous cell carcinoma by inhibiting AKT, a critical component of the mTOR pathway [67]. Furthermore, MiRNA-101 inhibits the progression of lung cancer by enhancing the expression of PTEN, suggesting that miRNAs − 101 may represent a novel potential therapeutic strategy in the treatment of lung cancer treatment [68]. The expression of galectin-1 (Gal-1) is associated with the migration and invasion of renal cell carcinoma (ccRCC) cells through the HIF-1α–mTOR signaling axis. overexpression of miRNAs − 22 inhibits the AKT/mTOR signaling pathway by targeting Gal-1. These findings show that targeting the miRNAs − 22/Gal-1/AKT/mTOR axis may be a potential therapeutic strategy for the treatment of ccRCC [69]. Similarly, overexpression of tumor suppressor miRNAs − 204 decreased the activity of mTOR and AKT downstream targets 4E-BP1 and S6K1 in cancer cells. Additionally, Loss of miRNA-204 induces the migration and invasion of cancer cells through activation of AKT/mTOR/Rac1 signaling and actin reorganization [70]. It has been shown that over-expression of miRNA-451 in colon cancer cells results in the reactivation of mTOR kinase activity by inhibiting the (AMPK), the negative regulator of mTORC1. Therefore, miRNA-451-regulated activation of mTOR activity facilitates colorectal cancer progression and may be a potential target in the treatment of colorectal cancer [71]. miRNA-mediated regulation of the mTOR signaling pathway is also related to enhancing chemosensitivity efficacy in cancer patients. Overexpression of miRNAs − 15a/16 or knockdown Rictor suppresses enhances chemotherapeutic effectiveness through downregulating mTORC1/p70S6K and promoting apoptotic cell death through excessive autophagy [72, 73]. miRNAs − 129-mediated suppression Notch1 promotes autophagic flux by suppressing mTOR activity and increasing Beclin-1 expression in glioma cells. Thus, miRNAs − 129 is a promising diagnostic marker and therapeutic target in glioma [74]. The mechanism of autophagy in tumor cells is the subject of a dispute. It is suspected that there is a limitation where autophagy could prevent cancer formation, while differently stimulating oncogenic progression [75].

### miRNAs regulating mTORC2

miRNAs could also influence cell survival, proliferation, and metabolism by modulating mTORC2 [12]. miRNAs regulate numerous malignancies by affecting the mTORC2 axis [76]. miRNAs − 153 could serve as a possible inhibitor agent which has a critical function in glioma cancer cells. Upregulation of miRNAs − 153 induces considerable suppression of cell proliferation and stimulation of apoptosis by modulating mTORC2 [77]. Moreover, miRNAs − 218 increases apoptosis and anti-tumor effects in cervical cancer cells via targeting Gli3 and IDO1 [78, 79]. However, miRNAs − 21 potentially increases cell proliferation by activating mTORC2 in renal cancer cells [80, 81]. It has been shown that miRNAs − 218 can promote osteoclastogenic differentiation by repressing NF-κB signaling which might be a therapeutic option [82]. In addition, Lin-4 and let-7 stimulate the mTORC2 network, which helps in inter-tissue transfer [83]. Taken together, these findings reveal that the miRNA-mediated regulation of mTORC2 is involved in tumor cell survival, and targeting these pathways could be a potential strategy for the treatment of different human cancers.

## Crosstalk between miRNAs and Cancer Progression during RT

miRNAs have emerged as influential regulators of gene expression, impacting various cellular processes and pathways crucial for tumorigenesis [84]. Their role in modulating tumor response to RT has sparked significant interest and exploration in the field of cancer treatment [85]. The intricate interplay between miRNAs and RT holds promise for enhancing therapeutic outcomes across different types of tumors [86].

### miRNAs and breast cancer

In the context of breast cancer, miRNAs have demonstrated their potential to modulate the cellular response to RT [87]. Certain miRNAs exert control over key proteins involved in DNA damage response and repair, thereby influencing the sensitivity of breast cancer cells to radiation [88]. Moreover, the dysregulation of specific miRNAs in breast cancer has been linked to radioresistance, highlighting the intricate involvement of miRNAs in shaping the response of tumor to RT [89].

### miRNAs and lung cancer

Moving to the realm of lung cancer, miRNAs have been identified as pivotal players in dictating the radiosensitivity of lung cancer cells. By targeting critical pathways involved in apoptosis, DNA repair, and cell cycle regulation, miRNAs can modulate the cellular response to radiation exposure. Furthermore, the dysregulated expression of certain miRNAs in lung cancer has been associated with radioresistance, underscoring the potential of miRNAs as determinants of treatment response in this context. Shifting focus to prostate cancer, the influence of miRNAs on RT response becomes evident [90, 91].

### miRNAs and prostate cancer

Understanding the impact of miRNAs on the androgen signaling pathway, DNA damage repair mechanisms, and tumor cell proliferation is essential in elucidating the intricate interplay between miRNAs and RT efficacy in prostate cancer. Furthermore, the identification of miRNA signatures associated with radioresistance provides valuable insights for refining treatment strategies and personalizing RT regimens for prostate cancer patients [92, 93].

### miRNAs and glioblastoma

In the context of glioblastoma, the unique challenges posed by this aggressive brain tumor underscore the importance of unraveling the role of miRNAs in RT response. MiRNAs have been implicated in regulating critical cellular processes such as angiogenesis, invasion, and stemness in glioblastoma, thereby influencing the response of tumor to RT. The intricate cross-talk between miRNAs and RT in glioblastoma underscores the potential for harnessing miRNAs as therapeutic targets to improve treatment outcomes [94, 95].

## miRNA-mediated regulation of mTOR signaling pathway and radiosensitivity

The targeting of mTOR by miRNAs has been shown to influence the radiosensitivity of cancer cells, suggesting a potential therapeutic strategy for enhancing the effectiveness of RT in cancer treatment. Several miRNAs, have been identified as regulators of mTOR signaling and have been found to modulate the response of cancer cells to radiation therapy. Understanding the specific miRNAs that target mTOR and their impact on radiosensitivity may provide new avenues for personalized cancer treatment approaches that can optimize the use of RT in individual patients.

### miRNAs − 21

Over the past decade, multiple miRNAs have been recognized as involved in radioresistance development in human papillomavirus HR-HPV-positive cervical cancer. However, the detailed regulative network of miRNAs in cancer radioresistance has remained to be elucidated. miRNAs − 21 is an essential miRNA that regulates the generation of radioresistance in HR-HPV-positive cervical cancer cells by inhibiting large tumor suppressor kinase 1 (LATS1) [96]. A recent finding has found that the viral oncoprotein E6 could enhance miRNAs − 21 transcription in cervical cancer [97]. Also, a connection between miRNAs − 21 upregulation and enhanced radioresistance has been discovered in certain cancers [98, 99]. Whereas the oncogenic effect of miRNAs − 21 in cancer pathology and its function in radioresistance progression has been established, how it is abnormally regulated in different radioresistant malignancies is still unclear.

miRNAs − 21 plays a critical role in regulating autophagy. In aggressive glioma cell lines, silencing miRNAs − 21 which is a well-known onco-miRNA in malignant glioma, increases autophagy activity through inhibition of the PI3K/AKT pathway and decreases radiosensitivity of cancer cells [100]. These findings reveal an important role of miRNAs − 21 in the radioresistance of malignant glioma and provide a novel therapeutic approach for improving the therapeutic efficacy of malignant glioma. Elevated expression level of miRNAs − 21 is associated with sorafenib resistance of hepatocellular carcinoma (HCC) cells by suppressing autophagy via the PTEN/Akt pathway [101]. Therefore, miRNAs − 21 could serve as a potential therapeutic target for overcoming sorafenib resistance in the treatment of HCC. Additionally, dysregulated autophagy is related to enhanced radiosensitivity in human cancers such as the nasopharyngeal carcinoma cell line [102]. mTOR inhibition via rapamycin diminishes radioresistance of radioresistant nasopharyngeal carcinoma (NPC) [103]. In comparison, miRNAs − 21 upregulation has the same result as 3-MA in suppressing autophagy whereas miRNAs − 21 suppression showed a comparable result in simulating autophagy such as rapamycin. According to a recent study, miRNAs − 21 reduced autophagy and enhanced radioresistance in siHa and Hela cells [103].

mTOR is a key modulator in the production of autophagy which can be stimulated through the PI3K/Akt pathway [104]. Also, the Akt-mTOR pathway promoted by miRNAs − 21, which is one of the crucial mechanisms of miRNAs − 21 facilitated autophagy suppression [103]. Upregulation of miRNAs − 21 is associated with HIF-1α overexpression in radioresistant cervical cancer. MiRNAs − 21 enhances the p-Akt, reduces PTEN, and subsequently increases HIF-1α expression. Therefore, there is a HIF-1α-miRNAs − 21 positive feedback loop through the PTEN/Akt/HIF-1α pathway in cervical cancer cells. MiRNAs − 21-mediated inhibition of PTEN results in increased mTOR signaling pathway and subsequently suppression of autophagy following irradiation. Therefore, miRNAs − 21 enhances radioresistance in cervical cancer cells by suppressing the autophagy [103]. Such mechanisms have been seen in hepatocellular cancer, where miRNAs − 21 is involved in the development of resistance to sorafenib by inhibiting autophagy via the Akt/PTEN pathway [100].

### miRNAs − 34

The tumor suppressor gene TP53 is one of the most frequently mutated genes in many types of human cancer. The production of the p53 protein, which is a major anti-tumor molecule, is controlled by a variety of transcription agents such as miRNAs − 34a [105]. miRNAs − 34a is committed to the suppression of oncogenesis, tumor metastasis, and reduction of radioresistance [106]. The transcription of miRNAs − 34a in cancer cells is at the minimum level, and miRNAs − 34a is associated with the RNA-induced silencing complex to control the activity of p53, which affects cell circle arrest, apoptosis, and DNA damage repair [107]. It has been shown that upregulation of miRNAs − 34a modulates the activity of the p53 through increasing p53 expression and decreasing the transcription of other proteins such as Sirtuin-1 (SIRT1)) [108]. Hence, cancer cell apoptosis was generated, and tumor cell development was suppressed. In addition, protein SIRT1 is engaged in the modulation of the PI3K/ PTEN/AKT network. FOXO1 and mTOR, which are essential proteins in the PI3K/PTEN/AKT network, were shown to be engaged in tumor cell progression, migration, apoptosis, and radiation resistance during RT [109].

The overactivation of the PI3K/AKT/mTOR signaling pathway is often found in human tumor tissues and is intimately associated with the generation of tumors [110]. It has been shown that The PI3K/AKT/mTOR network is involved in cell development, differentiation, and metabolism, as well as angiogenesis [111]. Also, miRNAs − 34a can considerably reduce the production of p-AKT/AKT and p-mTOR/mTOR. In addition, the inhibited PI3K/AKT/mTOR signal pathway is associated with the mechanism of the radiation resistance reversion effect of miRNAs − 34a [112]. The findings of xenograft trials confirmed that miRNAs − 34a into rECA-109 can increase the sensitivity of rECA-109 to radiation and result in a reduction in tumor development [112].

### miRNAs − 99

miRNAs could regulate radiation sensitivity by stimulating oncogenic pathways or decreasing tumor inhibitor gene pathways or protein production [113]. Changes in miRNA expression have been related to RT efficacy and could be employed as a prognostic factor for determining RT effectiveness [114]. For example, miRNAs − 99a has been discovered as a key prognostic component that affects radiation sensitivity in various malignancies, including prostate cancer [115]. miRNAs − 99a activity is increased in lung tumors when compared to normal cells of the same type [116]. These findings show that miRNAs − 99a could contribute to radiation sensitivity, but the process involved is still unclear [115]. Moreover, mTOR is involved in radiation sensitivity, and it has been demonstrated that inhibiting mTOR improves radiosensitivity [117, 118]. It has been demonstrated that inhibition of mTOR increased the radiosensitivity of some malignancies, such as lung cancer cells (103). Hence, it has been demonstrated that mTOR suppression, as a target of miRNAs − 99a, has the same effects on ectopic miRNAs − 99a production, but mTOR upregulation restores the activity of the miRNAs − 99a-mediated radiosensitivity [119]. Various essential functional modifications in genes, such as Bcl-2 and cyclin D1, which are associated with anti-apoptosis and the cell cycle, following miRNAs − 99a-mediated mTOR inhibition have been reported [120]. In particular, mTOR suppression via mTOR inhibitor could eliminate a contemporaneous behavior with the function of miRNAs − 99a and enhance radiosensitivity in apoptosis generation [119].

### miRNAs − 101

It has been shown that rapamycin could improve the radiosensitivity of lung cancer cells by suppressing mTOR and improving the radiosensitivity of human glioma stem cells [121, 122]. Furthermore, mTOR has been shown to regulate miRNAs − 101-3p in several cancer cells [123, 124]. These results mentioned that mTOR could be a possible oncogene that is associated with cell radiosensitivity. Additionally, miRNAs − 101-3p reversely modulates the activity of mTOR, suggesting that miRNAs − 101-3p could be essential for non-small cell lung cancer radiosensitivity [125]. The stimulation of mTOR signaling contains mTOR kinase and its subsequent receptor, ribosomal protein S6 [126]. Also, the development of mTOR pathway-related protein p-mTOR and p-S6 suppress with upregulation of miRNAs − 101-3p, which increases in the miRNAs − 101-3p depleted non-small cell lung cancer cells [125]. Hence, suppression of the mTOR signaling pathway improves radiosensitivity in radioresistant prostate cancer cells by suppressing colony formation, increasing apoptosis, and decreasing autophagy [127]. Also, rapamycin decreases mTOR function selectively [128]. For instance, rapamycin promotes radiation-induced apoptosis and promotes the destructive response of radiation in non-small cell lung cancer cells [122]. So, the inhibition of mTOR signaling increases irradiation sensitivity in the initiation of apoptosis, and miRNAs − 101-3p sensitizes A549 cells to irradiation by inhibiting the TOR [125].

### MiRNAs − 150

miRNAs − 150 has also been recognized as a crucial modulator of immune cell development and stimulation because it is predominantly generated in mature B and T cells, as well as NK cells [129]. miRNAs − 150 has been identified as a tumor inhibitor in various human cancers [130, 131]. Although a previous report has found that miRNAs − 150 was attenuated in NK/T cell lymphoma, the function, and processes of miRNAs − 150 in this malignancy remain unknown [132]. Previously reported reduction in miRNAs − 150 in NK/T cell lymphoma biopsies [132]. Based on these findings, reduced amounts of miRNAs − 150 have been related to some aggressive aspects of NK/T cell lymphoma, including EBV viral load and aggressive lymphoma to EBV [133]. Upregulation of miRNAs − 150 might result in EBV-positive lymphoma progressing to a more advanced stage [134]. Furthermore, reduced miRNAs − 150 activity in NK/T cell lymphoma is associated with poor treatment outcomes. As a result, the miRNAs − 150 expression might be a helpful supplementary agent to guess the NK/T cell lymphoma treatment outcome [135].

So far, multiple miRNAs NAs have been identified in contributing to modulating therapeutic efficacy in cancer treatment [136, 137]. A preclinical study has shown that miRNAs − 150 can increase the sensitivity of NK/T cell lymphoma cells to radiation [135]. The achievement of satisfactory outcomes during RT is related to its capacity to induce apoptosis in tumor cells [135]. Regarding this, The PI3K/AKT mechanism could cause cancer cells to become resistant to RT, and suppression of this mechanism could improve their susceptibility to therapy [138, 139]. Also, the miRNAs − 150 modulation pathway is associated with the activation of AKT [140, 141]. Upregulation of miRNAs − 150 suppresses the PI3K/AKT/mTOR signaling pathway, and PI3K tyrosine kinase inhibitors improve miRNAs − 150 activities, demonstrating that the PI3K/AKT/mTOR signaling pathway is essential in miRNAs − 150-mediated radiosensitivity. Overexpression of miRNAs − 150 could suppress the PI3K/AKT/mTOR signaling pathway, and the PI3K tyrosine kinase inhibitor enhances the effects of miRNAs − 150, indicating that the PI3K/ AKT/mTOR signaling pathway is involved in miRNAs − 150 mediated radiosensitivity [135].

### miRNAs − 410

PTEN is a well-known inhibitor of the PI3K/Akt signaling pathway [142]. PTEN deficiency in non-small cell lung cancer results in increased PI3K/ AKT signaling pathway and downstream mTOR, which affects a variety of cellular activities [143]. According to previous findings, PTEN might involve non-small cell lung cancer epithelial-mesenchymal transition and radioresistance [144]. Moreover, increasing data suggest that stimulation of the PI3K/Akt/mTOR pathway might trigger the epithelial-mesenchymal transition, and it serves a crucial function in radioresistance downstream of the epidermal growth factor receptor pathway [145]. Dual suppression of PI3K/AKT/mTOR improves radioresponse via modulating the DNA damage response mechanism [146]. Likewise, the upregulation of miRNAs − 410 in non-small cell lung cancer cells has been observed in the amounts of phosphorylated Akt, mTOR, P70S6K, and 4E-BP1 [147]. On the other hand, the suppression of miRNAs − 410 suppresses the AKT/mTOR pathway. A specific PI3K, Akt, or mTOR suppressor drastically reduces miRNAs − 410-induced epithelial-mesenchymal transition and radioresistance in PC9-miRNAs − 410 and SPC-A1-miRNAs − 410 cells [147]. PTEN activity in PC9-miRNAs − 410 and SPC-A1-miRNAs − 410 cells reduces miRNAs − 410-induced EMT and radioresistance, but siPTEN transfection in A549-Inh and H1299-Inh cells causes apoptosis. So, the PTEN might be required for miRNAs − 410-induced PI3K/mTOR upregulation, promoting both epithelial-mesenchymal transition and radioresistance [147, 148]. MiRNAs − 410-3p has been shown to have oncogenic activities in prostate cancer through the PTEN/AKT/mTOR pathway [149]. Also, another study found that reduction of the lncRNA OIP5-AS1 caused miRNAs − 410 to accumulate and modulate its target KLF10/PTEN/Akt-mediated cellular activities [150]. In addition, these findings revealed a connection between miRNAs − 410 and the PTEN/Akt/mTOR axis in cancers.

It has been revealed that in SPC-A1 miRNAs − 410 cells, increasing production of phosphorylated PI3K/mTOR pathway indicators correlates with increased amounts of mesenchymal markers, while E-cadherin and PTEN levels reduce remarkably [147]. Furthermore, SPC-A1-miRNAs − 410 cancer cells are much more radioresistant, evidenced by a shorter proliferation delay and lower amounts of -H2AX. Therefore, miRNAs − 410 upregulation might promote the epithelial-mesenchymal transition mechanism and radioresistance, connected to the PTEN/PI3K/mTOR pathway [147, 148]. Based on these findings, miRNAs − 410 could appear to be a promising target to increase the radioresponse of non-small cell lung cancer.

### miRNAs − 519

**Fig. 3:**
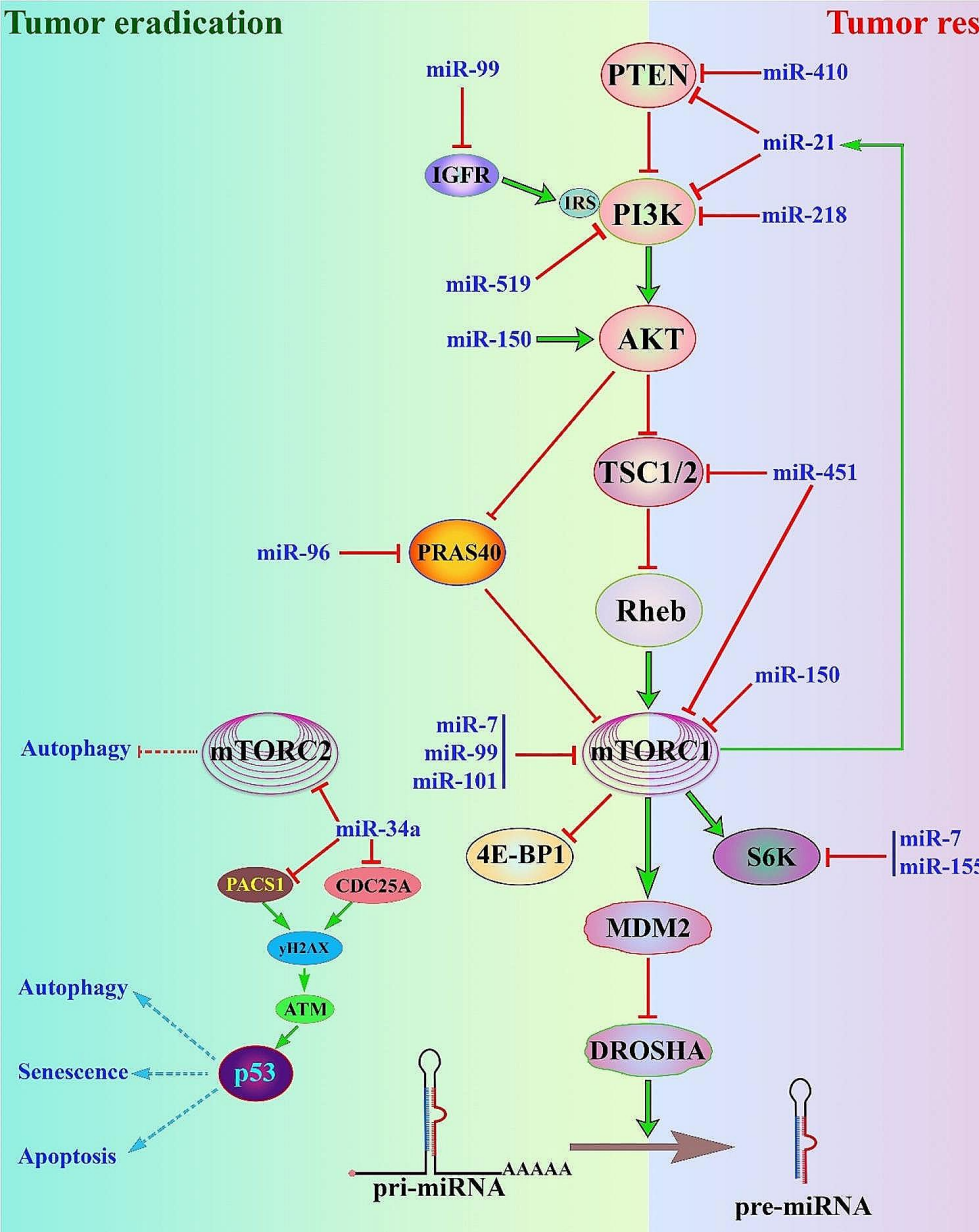
mTOR signaling-related MicroRNAs and Cancer involvement

MiRNAs − 519 is a known tumor inhibitor that has been found in a variety of cancers, such as nasopharyngeal carcinoma, colorectal cancer, and cervical cancer [151, 152]. According to the most recent data, miRNAs − 519 expression in esophageal squamous cell carcinoma tissues was lower than in non-cancerous tissues [153, 154]. Furthermore, the number of miRNAs − 519 indicates a lower overall survival rate in esophageal squamous cell carcinoma patients, as well as those who have had RT. The previous study has shown that the upregulation of miRNAs − 519 reduces the amounts of p-PI3K, p-AKT, and p-mTOR [155]. In addition, the administration of 7 a PI3K agonist 40Y-P altered the cell proliferation capacity and apoptosis induced by miRNAs − 519 overexpression in esophageal squamous cell carcinoma cells challenged with irradiation [155]. Based on this study, the stimulating effect of miRNAs − 519 in the radiosensitivity of esophageal squamous cell carcinoma patients was clarified, and a new promising biomarker for RT was suggested (Fig. 3).

## Nanotechnology for miRNAs delivery

Nanotechnology can create new resolutions to improve mRNA interference treatment and overcome the challenges ahead for RT treatment for the therapeutic use of mRNAs. Many nanocarriers including liposomes, polymerosomes, micelles, and metallic nanoparticles have been applied for mRNA loading or attachment for different applications [156]. Nanotechnology has been applied for mRNA detection [157] or mRNA interference therapy [156]. Many nanostructures have been applied to increase the efficacy of the radiation treatment. This includes metallic nanostructures such as gold [158], iron [159], or drug carriers such as vesicles [160] or mesoporous silica [161].

Also, various nanoparticles have been applied for MTOR targeting [162]. However, in most of the studies that have targeted the mTOR, no nanocarriers have been applied to deliver mRNAs. Due to the many problems of using mRNA compounds in human studies or even in in-vitro or in-vivo experiments, nanoparticles can be used for mRNA delivery.

The EnGeneIC delivery vehicle (EDV) is a biologically derived 400 nm particle that has been acquired from *Salmonella typhimurium* [163]. EVDs have been applied for encapsulation of miRNAs − 16 for mesothelioma delivery [164, 165]. The miRNAs − 7 was also loaded in EVDs and targeted for adrenocortical carcinoma tumors. It has been demonstrated that nanotechnology-based miRNAs − 7 treatment leads to overexpression of the mechanistic target of rapamycin (MTOR) which results in inhibition of CDK1 [166]. Mokri et al. have synthesized the folic acid functionalized chitosan zinc-based metal-organic framework nano complex, which has been loaded with miRNAs − 224 to target BECLIN1, mTORC1, and Caspase-9 [167]. The chitosan coating provides the necessary positive charge for miRNAs − 224 loading and the folic acid could enhance the cell internalization of the nanosystem.

One potential avenue lies in the utilization of exosomes as carriers for miRNA delivery. Exosomes are nanosized vesicles secreted by various cell types and have garnered attention for their potential as natural delivery vehicles for therapeutic molecules, including miRNA [168]. These naturally occurring vesicles possess intrinsic properties that make them particularly well-suited for miRNA delivery, such as stability in biological fluids, ability to cross biological barriers, and low immunogenicity [169]. Furthermore, advancements in bioengineering have enabled the modification of exosomes, allowing for the precise loading of therapeutic miRNA and enhanced targeting to specific cell types or tumor microenvironments. By harnessing the natural cell-to-cell communication mediated by exosomes, researchers can potentially optimize the delivery of miRNA to target cells, thereby augmenting the therapeutic impact of RT [170, 171].

In addition to exosomes, the development of sophisticated nanocarriers tailored for miRNA delivery holds promise for improving RT outcomes [172]. These nanocarriers can be engineered to protect miRNA from degradation, evade immune detection, and facilitate targeted delivery to tumor sites [173]. Moreover, the integration of stimuli-responsive materials within these nanocarriers enables controlled release of miRNA in response to specific physiological triggers, thereby enhancing precision and efficacy. It is important to note that the field of miRNA delivery for RT is dynamic and rapidly evolving, with diverse strategies being explored to overcome existing challenges. These include the refinement of physical methods such as ultrasound-mediated delivery, the application of viral vectors, and the exploration of combination therapies that synergistically enhance miRNA delivery and RT efficacy [173, 174].

## Clinical translation

Due to the newness of this research field, clinical studies have not yet started in this field. According to pre-clinical studies, the clinical translation of miRNAs could target mTOR as therapeutic agents to improve RT outcomes holds promise for enhancing the efficacy of cancer treatment. In recent studies, miRNAs such as miRNAs − 100, miRNAs − 99a, and miRNAs − 21 have demonstrated their ability to regulate mTOR signaling and influence the radiosensitivity of cancer cells, providing a potential avenue for therapeutic intervention [175, 176]. The identification and understanding of specific miRNAs that target mTOR and their impact on radiosensitivity could pave the way for personalized cancer treatment approaches that optimize the use of RT in individual patients. By targeting mTOR, these miRNAs may help overcome radioresistance in cancer cells, leading to improved treatment outcomes and potentially reducing the risk of tumor recurrence. Further research and clinical trials are needed to validate the efficacy and safety of using miRNAs targeted at mTOR as therapeutic agents in combination with RT, ultimately translating these findings into improved clinical outcomes for cancer patients.

## Conclusion and future aspects

Nowadays, RT is considered a successful strategy in the treatment of various malignancies. Nevertheless, following several doses of radiation, radiation resistance will be the primary reason for RT failure. mTOR expression and activation by radiation play a critical role in radioresistance during RT. Recently, miRNAs have developed potential predictive and diagnostic indicators, including therapeutic candidates for innovative and customized cancer treatment. Various miRNAs have been recorded differentially produced and are prognostic of treatment outcomes in a variety of cancers. In the current review, we highlighted the impact of several miRNAs targeted mTOR in radioresistance and radiation-induced changes to overcome the limitation of RT, which could provide a way for customized therapy in the future. As a future aspect, nanotechnology can play a crucial role in the development of efficient delivery systems for miRNAs targeting mTOR to enhance RT outcomes. Nanoparticles can be designed to encapsulate and protect miRNAs, allowing for targeted delivery to cancer cells and minimizing off-target effects. Additionally, nanocarriers can be engineered to release miRNAs in a controlled manner, ensuring sustained and optimal therapeutic effects.

## Data Availability

No new data generated by this study.
