# Correction: MicroRNAs targeted mTOR as therapeutic agents to improve radiotherapy outcome

**DOI:** 10.1186/s12935-024-03461-8

**Published:** 2024-08-03

**Authors:** Shahram Taeb, Davoud Rostamzadeh, Seyed Mohammad Amini, Mohammad Rahmati, Mohammad Eftekhari, Arash Safari, Masoud Najafi

**Affiliations:** 1 Department of Radiology, School of Paramedical Sciences, Guilan University of Medical Sciences, Rasht, Iran; 2 Department of Immunology, University of Connecticut Health Center, Farmington, CT USA; 3 Radiation Biology Research Center, Iran University of Medical Sciences, Tehran, Iran; 4 Department of Medical Biotechnology, Faculty of Paramedicine, Guilan University of Medical Sciences, Rasht, Iran; 5 Ionizing and Non-Ionizing Radiation Protection Research Center (INIRPRC), School of Paramedical Sciences, Shiraz University of Medical Sciences, Shiraz, Iran; 6 Department of Radiology, School of Paramedical Sciences, Shiraz University of Medical Sciences, Shiraz, Iran; 7 Radiology and Nuclear Medicine Department, School of Paramedical Sciences, Kermanshah University of Medical Sciences, Kermanshah, Iran; 8 Medical Biology Research Center, Institute of Health Technology, Kermanshah University of Medical Sciences, Kermanshah, Iran; 9 Medical Technology Research Center, Institute of Health Technology, Kermanshah University of Medical Sciences, Kermanshah, Iran

**Correction to: Cancer Cell International (2024) 24:233**

10.1186/s12935-024-03420-3

In this article [1], the affiliation details for the author Arash Safari were incorrectly given as ‘Department of Radiology, Ionizing and Non-Ionizing Radiation Protection Research Center (INIRPRC), School of Paramedical Sciences, Shiraz University of Medical Sciences, Shiraz 71439 − 14693, Iran’ but should have been ‘Ionizing and Non-Ionizing Radiation Protection Research Center (INIRPRC), School of Paramedical Sciences, Shiraz University of Medical Sciences, Shiraz, Iran and Department of Radiology, Paramedical School, Shiraz University of Medical Sciences, Shiraz, Iran’.
